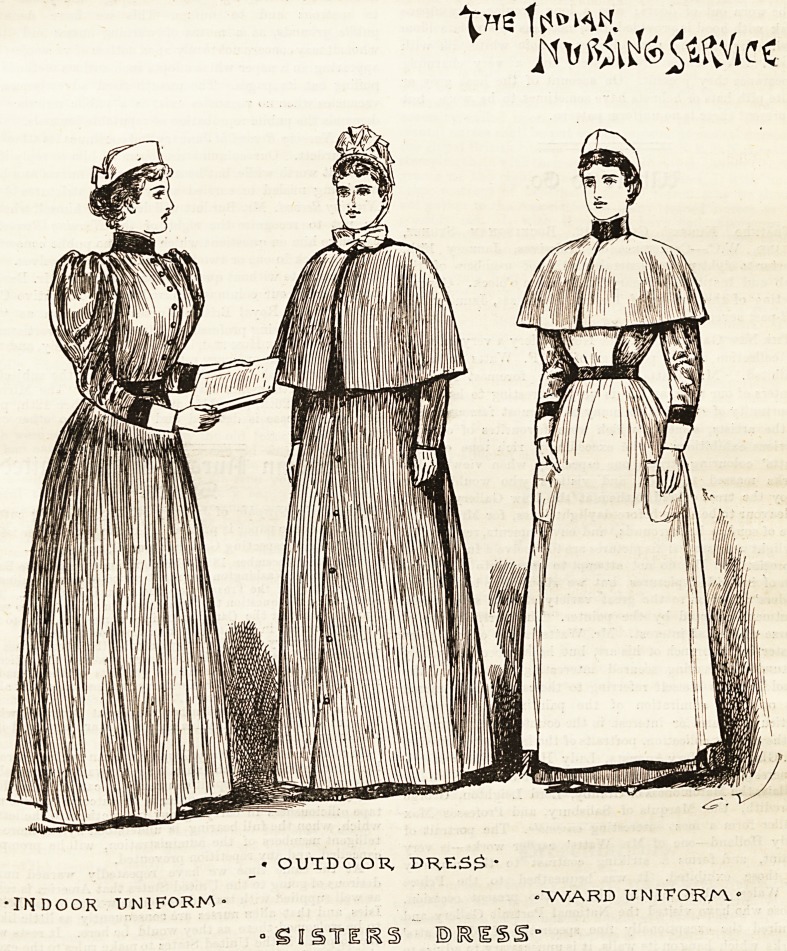# "The Hospital" Nursing Mirror

**Published:** 1897-01-09

**Authors:** 


### "The Hospital" Nursing Mirror.

Being the Nursing Section of "The Hospital."

[Contributions for this Section of "The Hospital" should be addressed to the Editor, The Hospital, 28 & 29, Southampton Street, Strand, London, W.C., and should have the word "Nursing" plainly written in left-hand top corner of the envelope.]

### News from the Nursing World.

### THE ROYAL NATIONAL PENSION FUND FOR NURSES.

The secretary of the Pension Fund desires us to inform policyholders through our columns that they can send their contributions to the Junius S. Morgan Benevolent Fund to him annually, as suits their convenience, but in a sum amounting to not less than one shilling. He particularly requests that nurses will mark letters or envelopes "Benevolent," as money has reached him without any indication as to its intended destination.

### CHRISTMAS TREES AT THE LONDON HOSPITAL.

"Christmas Tree Day" is always a great occasion for the gathering together of "old Londoners," who come from far and near to see the children in Queen and Beatrice wards enjoy the time-honoured Punch and Judy shows and the gift-laden trees, and to greet friends in the hospital world whom they see at no other time. This year was no exception to the rule; more visitors than usual thronged the hospital throughout the afternoon of December 28th, and the newly-opened wing of the Nurses' Home was an additional attraction which aroused much admiration. This new building provides seventy-five more bed-rooms, each of them charming little apartments, amply and prettily furnished. It is difficult to understand why it has not been possible to connect the two wings of the Home together on each floor, for, as it is, nurses working in the top wards of the hospital have to descend to the very ground floor, from which alone can access be had to the new wing, and should their rooms be on the third or fourth floor, the journey is one of no inconsiderable length. The room in the old Home formerly apportioned to the sisters' use has been thrown into the nurses' dining-room, and the addition of a delightful new sick-room places the old one at liberty for use as a sisters' dining-room. Amongst the visitors on Monday were: the Hon. Sydney Holland, the new chairman of the hospital, who was accompanied by Lady Knutsford; Lady Dorothy Nevill, and many members of the honorary medical staff of the hospital and well-known London matrons and superintendents; amongst them Miss Gordon (St. Thomas's), Miss H. Gordon (Charing Cross), Miss Bland (Poplar), Miss Robinson (Moorfields), Miss Gray (Bloomsbury), and Miss Philippa Hicks (Nurses' Co-operation).

### ROYAL BRITISH NURSES' ASSOCIATION AND MENTAL NURSES.

The proposal to admit mental nurses or asylum attendants on the register of the R.B.N.A. has caused a good deal of searching of heart amongst some of its members, who do not approve of the scheme. Miss Sophia Wingfield, late matron of the General Infirmary, Macclesfield, a member of the General Council of the Association, is organising a protest against what she considers to be a "degradation of the register," and a meeting on the subject was held at St. Martin's Town Hall, Charing Cross, on January 7th, to which "trained nurses and members of the general public whose interests are at stake" were invited.

### HELP FOR THE INCURABLES.

The Board of Management of the British Home for Incurables, Streatham, is appealing for 500 new annual subscribers of £1 1s. each, to enable them to fill the beds in the home at Streatham. There are now 65 patients; it was planned to accommodate 70, but the furnishing of some of the rooms is not yet complete, owing to lack of money. Including 300 partially disabled annuitants, altogether, on an average, the sum of £30 a day is given through this charity to sufferers from incurable disease in all parts of the United Kingdom. Who will assist in providing a day's expenditure? Cheques and postal orders to be crossed "Barclay and Co., Limited," and sent to the secretary, Mr. R. G. Salmond.

### NURSING IN IRISH WORKHOUSES.

A conference of the Irish Workhouse Reform Association will be held in Dublin on the 28th inst., at which the Earl of Meath is expected to take the chair, and the question of nursing is one which will come uppermost for discussion. Gertrude, Countess of Pembroke, has generously offered to train twelve nurses for workhouse duty, to be called the Pembroke Nurses and be placed at the service of Guardians throughout the various counties of Ireland. Arrangements have already been made for entering two probationers at the City of Dublin Nursing Institution, 27, Upper Baggot Street, where they will be trained in the wards of the City of Dublin Hospital under the conditions of the Nursing Institution. It is proposed that three others shall be placed for instruction at the North Dublin Union, under Miss West, the superintendent there.

### NURSING AT THE CAPE.

We have often warned nurses who are anxious to try their luck in strange places that it is well to make sure before starting of the sort of work they are about to undertake. The following paragraph in the *Cape Argus*, December 3rd, 1896, points the same moral: "Johannesburg, December 2nd [Special].—The Hospital Board has sued three nurses for £100 each for breach of contract. The defendants were brought out from England on an agreement which stipulated that they would receive the same treatment and hours as in London, and if any of them left the hospital before the expiry of three years they would pay £100 to the Board as liquidated damages. According to the evidence for the defence, the nurses were badly treated, had miserable rooms, bad food, very long hours, and for the first month had not a single day off, the only time allowed being two hours a day, yet the Board said they ought to be ashamed of themselves. No lady could stand the treatment they received, which was the reverse of that experienced in England. A double staff was necessary Judgment was given against the defendants, with costs. An appeal was noted."

### THE FLANNEL SHIRT CLUB.

A new club, with the very excellent object of supplying flannel shirts to men patients leaving hospital without proper warm clothing, has been started under the above title, and the first meeting was held at the house of the president, the Countess of Strafford, on December 16th. The idea is a very happy one, for the useful but uninteresting shirt is just the article that people do not include in their bundles of dainty women's and children's garments, petticoats, bed jackets, &c., which are so pleasant and easy to make, and for which there is an unlimited demand, too. But the sisters of male wards have sad stories to tell of the dearth of "men's things," and to them the thought of the good things to come from the "Flannel Shirt Club" will be a most comforting one. Mrs. W. L. Courtney is vice-president, and Mrs. H. H. Chilton, hon. treasurer. All particulars of the society's rules can be had from the hon. secretary, Miss Lamport, 52, St. John's Wood Road, N.W.

### CHRISTMAS APPEALS.

A charming little reminder of the needs of the child patients has been sent out this year in the shape of a card "from the sick children in the North-West London Hospital, with their love and thanks for all you have done for them, and wishing you a merry Christmas and a happy New Year." The pretty picture of a child with a cat in her arms, "Pussy must go to the Hospital," was engraved and the card supplied free of cost by the Strand Engraving Company.—An appeal which should touch the hearts of the hardest has gone forth this Christmas from the Victoria Hospital for Children, Chelsea, in the form of a tiny booklet, "An Angel in the Ward." It is on behalf of the Gabriel Ward for Infants, hitherto supported by a special subscription of £400 a year, now, alas! to be discontinued, and the making up of this deficiency is a serious matter. The plea for the tinies is most touchingly written, and should bring a ready response.

### ST. CATHERINE'S HOME FOR INCURABLES, BRADFORD.

It is pleasant indeed to be able to congratulate the committee of this useful and deserving institution upon the generous help just offered to them by Mr. Cawthra, of Horton Hall, Bradford. An extension of the home has been a crying need for some time, and a fund has been opened towards this object, to which kind friends had contributed nearly £800 in the first week. Then, in response to an appeal for help, came from Mr. Cawthra (who had been intending to present some gift to the town of Bradford, and has made an excellent choice in deciding to make it through St. Catherine's) an offer to purchase a site and build at his own expense a new home. The ground is already secured, and plans are being prepared; while the money received for the purposes of extension is, with the ready consent of the donors, being transferred to an endowment fund, which the committee hope to raise to £2,000. It may be imagined that gratitude and thankfulness at the hopeful prospect before them are reigning amongst the many friends of St. Catherine's this Christmas.

### NURSING IN IRELAND.

The directors of the City of Dublin Nursing Institution have resolved to raise the salary of the lady superintendent, Mrs. J. Kildare Tracey, by £20 per annum. Mrs. Tracey is the widow of a County Kilkenny gentleman, and received her training in the wards of the City of Dublin Hospital, where she remained a member of the nursing staff for several years, until, in 1890, she was appointed to succeed the late Miss Fitzgerald in her present responsible post. The nursing institution is an establishment distinctly apart from the hospital, although probationers are trained in and nurses supplied to the latter by the former. It has a board of management of its own, defrays its own expenses, and maintains its own members. There are at present one hundred and twenty nurses employed by the institution, some at the private hospital, Mespil House, others at the branch establishment at Pau, and a great many engaged in private nursing throughout Ireland.

### NURSING IN PRISON INFIRMARIES.

We have been asked by several correspondents if trained nurses can secure appointments in prison infirmaries as matrons or nurses. We understand that they can only do so by applying for the position of assistant warder, in which capacity they would have to perform all the usual duties devolving on such officers. Attendance in the infirmary ward would only be undertaken when required as part of their usual routine. No person, even in the largest prisons, is employed solely in the care of the sick. The infirmary for female prisoners is entirely the care of the head matron, who employs her assistant warders in ministering to the invalids as she sees best from day to day. It is an advantage to such officers to have had hospital training, but they are never appointed for that reason only. If necessary, a temporary warder is sometimes engaged from outside the prison. The infirmaries of the male prisoners are entirely served by men—the regular warders—under the direction of the medical officer, who is, of course, equally paramount on the women's side. Any trained nurse or other person wishing to obtain a situation as assistant warder should apply at the nearest prison for a printed form of application, which she will have to fill up and return to the governor; he will then forward it to the Prison Commissioners at the Home Office, with whom alone the decision will rest as to whether the applicant is eligible or not.

### SHORT ITEMS.

At the Field Lane Refuges excellent dinners have been given this Christmas and New Year to many hundreds of destitute men, women, and children.—The Charlotte Home for Invalid Children, recently opened at Bournemouth by Mr. J. H. Shayler, kept the first Christmas of its existence in festive fashion; entertainments were given to the children, and they were made happy by many gifts of clothing, toys, sweets, and crackers from kind friends in the neighbourhood. —We announced last week that Miss Lynch had been appointed Superintendent of Nurses at the Ophthalmic School, Hanwell, instead of superintendent nurse, as we should have stated.—We are sorry to learn from Miss Vacher, who was appointed to the Matronship of the Kimberley Hospital in 1895, that she has been obliged to resign her post on account of ill-health and give up nursing altogether.—The marriage of Nursing Sister Aird with Surgeon-Captain Barnett-Wilson took place at Roorkee (the residence of the bride's sister) on December 3rd, 1896. The bride and bridegroom afterwards departed for Hurdwar. Among the presents was a handsome case of silver fish knives and forks, bearing the inscription: "To Sister Aird, from her grateful patients in the 1st Norfolk Regiment."

### Hygiene: For Nurses.

By JOHN GLAISTER, M.D., F.F.P.S.G., D.P.H.Camb., Professor of Forensic Medicine and Public Health, St. Mungo's College, Glasgow, &c.

### XXXVIII.—DISINFECTION — NATURAL DISINFECTANTS — PRACTICAL DISINFECTION OF EXCRETORY PRODUCTS, OF PATIENT, OF CLOTHING, AND OF ROOM.

SOMETHING now remains to be said regarding natural disinfectants, or germicides. It is very probably true that in our anxiety to accomplish the destruction of micro-organisms by artificial means we have somewhat overlooked those natural processes which effect the same end, and in a kindred fashion. Considering the omnipresence of disease-producing micro-organisms, the wonder is, not that these diseases are produced, but that they prevail in so light a manner, speaking comparatively. It is undoubted that this is brought about in large degree by the effects of air and sunlight in destroying such microbes. Of air as a germicidal agent it may at once be said that when fresh and abundant it so dilutes and oxidises infective matter as to reduce the virulency of these organisms, if not, indeed, to destroy them. The presence of ozone in the air materially assists such oxidation processes, but, unfortunately, it is but rarely to be found in the air of populous places. It is well known that micro-organisms are found in fewest numbers out at sea, and, doubtless, this is owing in large part to the existence of ozone. As has been already shown the area of infectivity of certain diseases is limited by the presence of air, and for the reason that the oxygen of the air renders the infective matter inert. This is notably the case in typhus and some other diseases. The anærobic forms of microbes cannot exist alongside of air, while the ærobic forms cannot exist without it. The microbe of tetanus is an example of the former class, that of diphtheria of the latter. Light is a valuable adjuvant to air in microbe destruction. Experiments have proved that exposure to light is fatal to several microbes, notably those of erysipelas, enteric fever, and others; while exposure to sunlight destroys that of tubercle in a few hours, and that of anthrax in a short time. If others be not actually destroyed, their vitality and virulency are at least hindered. These facts teach the necessity for good ventilation and lighting of our homes, and indicate the value of open spaces, good building regulations, and the prevention of smoke-production in our populous places.

The practical application of the principle of disinfection next falls to be considered. Given a patient suffering from an infective disease in a sick-room, the process of disinfection must not only be concurrent with the illness, but it must succeed the vacation of the room by the patient. As a leading, guiding, and safe principle, it may be said that everything that proceeds from the body of such a patient during the currency of the illness must be deemed infective, and be accordingly disinfected.

Let us consider in some detail the discharges from each disease, which demand disinfection.

SMALL-POX.—All discharges infective. Nasal and throat discharges to be received on cotton or linen rags, and burned. Stools, vomited matters and urine, to be disinfected with one pint of 4 per cent. solution of chloride of lime, or a like quantity of 1 to 1,000 solution of corrosive sublimate. The virus being concentrated in the skin eruption, the pustules ought to be daily coated with benzoated lard or eucalyptolised vaseline, especially during the "scabbing" stage. Crusts, when shed, to be carefully burned.

CHICKEN-POX.—As in small-pox.

SCARLET FEVER.—In acute stage, throat and nasal discharges to be treated as above. In "peeling" stage, body of patient to be sponged daily with equal parts of sanitas and tepid water, and to be washed twice weekly, at least, with carbolised soap and warm water. Special attention to be paid to toilet of hair, ears, and flexures of limbs.

MEASLES.—In acute stage, throat, nasal, and bronchial discharges to be treated as above. In convalescent stage, skin, as in scarlet fever.

GERMAN MEASLES.—As in ordinary measles.

TYPHUS FEVER.—All discharges infective. To be treated as above, in respect of skin, and other bodily products.

ENTERIC FEVER.—Stools chiefly infective, and must be disinfected with solution of chloride of lime or corrosive sublimate of above strengths. Carbolic acid is not an efficient disinfectant of the bacillus of this disease, unless used very strong.

CHOLERA.—To be dealt with as in enteric fever.

INFLUENZA.—Generally as in measles, especially as to mucous discharges.

WHOOPING COUGH.—Nasal, throat, and bronchial discharges, chiefly contain infective matter. To be treated as above.

DIPHTHERIA.—Nasal and throat discharges, chiefly infecfective, ought to be burned.

ERYSIPELAS.—Chiefly in skin; disinfection accomplished usually if iodine treatment be adopted. "Peeled" skin to be burned.

PUERPERAL FEVER.—Specially located in discharges from genital tract; ought to be received on antiseptic wood-wool pads, and burned; local antisepsis and lavements under advice of physician; other discharges disinfected with solution of corrosive sublimate.

TUBERCULAR PHTHISIS.—Sputum to be received on paper, or rags and burned, or into a spittoon containing sawdust, and burned.

In order to properly disinfect solid, semi-solid, or viscous discharges it is necessary either to mix the disinfectant with the discharge mechanically, or allow the disinfectant to soak for at least half an hour, in order that germicidal action may the better operate, before emptying into the slop or water-closet, which, after flushing, ought each time to be subsequently disinfected. The quantities and strengths of disinfectants to be used have already been indicated.

When the period of infectivity is safely over, the convalescent patient must be transferred into another room, in order that the apartment he has quitted, and its contents, may undergo disinfection. If care has been exercised during the illness, little risk to the other members of the household is likely to happen. But immediately prior to removal, the patient must be washed all over, and after having been carefully dried, ought to be wrapped in a fresh warmed blanket, which has been handed into the room just when needed. He should then be received by another person at the door of the room, and conveyed to his new apartment, which previously has been suitably warmed, to prevent chills. In scarlet fever, and in diphtheria especially, the patient should continue to use for some time after apparent recovery some antiseptic gargle.

The room just vacated and its contents now demand attention. The clothing which the patient has left behind, that of the nurse, bed-clothing and room-furnishings, must be disinfected either *in situ*, or be removed by the sanitary authority for that purpose. In rural and smaller urban districts, where no provision is made for this by the sanitary authority, the disinfection must be dealt with by the householder. The plan to carry out this is as follows: Articles of clothing, bed-clothing, &c., should be steeped in the room for a couple of hours in a solution of 1 to 2,000 corrosive sublimate, should then be roughly wrung, and, in a covered vessel, be removed to the wash-house, to there undergo the usual cleansing process, after which they should be hung in the open air, or laid upon the grass for 24 hours. Mattresses, pillows, &c., should be freely sponged with the same solution, removed to a place at some distance from the house, and then left exposed to the air, under cover if need be, for several days. All old articles may be burned, and in smallpox ought to be, whether old or new.

The wood-work, floor, and furniture of the room, the walls and ceiling, still remain to be disinfected. The former are usually left until the air of the room is disinfected. This

FIG. 1.

so-called aërial disinfection is an entire misnomer; indeed, at best, it is but a delusion. If ventilation during the currency of the illness has been attended to, little fear need be entertained of its infectivity. What do require attention, however, are the microbes and their spores, which by this time have become constituent parts of the dust which has settled in the crevices and corners and other points of lodgment in the room. In other countries than ours little or no attention is given to aërial disinfection, while much has been paid to the deposited dust. Hence, two main plans of room-disinfection prevail; one, devoted mainly to aërial disinfection as in our own country; the other, devoted entirely to disinfection of room-dust, chiefly in France and Germany. Each plan demands some explanation.

The English Local Government Board recommends that $1\frac{1}{2}$ lbs. of sulphur, mixed with a liquid combustible, as alcohol —some add nitre— should be burned for every 1,000 cubic feet of air-space in a room (which has been made as air-tight as possible), and the resultant sulphurous acid gas be allowed to operate for 12 to 24 hours It has, however, been clearly pointed out that this amount of sulphur only gives in the space named 1·75 per cent. of gas, whereas 4 per cent. is necessary for bactericidal effect. Apart from this, however, the burning of sulphur is by no means an easy matter. This uncertainty of action is easily overcome, if, instead of solid sulphur, cylinders or siphons of compressed liquid sulphurous acid gas are used. Fig. 1 shows such a cylinder, and Fig. 2 a siphon.

FIG. 2.

The gas is compressed at a pressure of about 45 lbs. per square inch, and when a cylinder is to be used all that is necessary to liberate the gas is to cut the projecting soft lead pipe with a knife.

Before using sulphurous acid gas, however generated, and in order to render it more efficient in action, the walls and ceiling of the room ought to be moistened with water, otherwise much of its potency is lost.

Instead of this gas chlorine gas is sometimes substituted. It is even more effective than the former, but it acts corrosively on metallic substances, and its fumes are exceedingly pungent and irritating. Doubtless, when it can be put up in the compressed liquid form, its use will revive because of its greater potency.

---

### The Royal National Pension Fund for Nurses.

### THE JUNIUS S. MORGAN FUND.

THE first ceremony in commemoration of the sixtieth year of Her Majesty's reign took place at Sandringham on January 1st, when the Prince of Wales, as patron of the Royal National Pension Fund for Nurses, presented the president, the Princess of Wales, with (1) the sum of £2,500 collected by the policyholders of the Fund, with (2) £2,500 presented by Mr. J. Pierpont Morgan and Mr. and Mrs. W. H. Burns, and (3) with an annual income of about £100 in annual subscriptions of 1s. and upwards given spontaneously by the nurse members. It is a gratifying and historical fact that the first of Her Majesty's subjects have been the first to commemorate the sixtieth year of her reign by taking part in a work connected with the Junius S. Morgan Benevolent Fund, the objects of which are to afford immediate pecuniary or other relief by loan or absolute gift to members of the Pension Fund who may be in distress and to assist them in keeping up their premiums. No class of Her Majesty's subjects do better work than the really competent nurses who minister to the sick throughout her dominions, and it is gratifying to know that the Junius S. Morgan Benevolent Fund has thus been made adequate to meet the claims upon it. The whole of the money presented to the Fund through the Princess of Wales has been provided so promptly and quietly, free from the usual expenses of organisation, that this act of commemoration was fittingly concluded by the ceremony at Sandringham on January 1st last.

We at first contemplated publishing a complete list of the 700 nurses by whose exertions the £2,500 was raised in donations. It appears, however, that the majority of them object strongly to their names being published, and so we have abandoned the idea. It cannot fail to interest all who are connected with the Pension Fund to know that among the collectors were policyholders at Kimberley, Durban, and the Cape, who collectively raised £10; in the United States of America at New York and Kansas City; at Vancouver, British Columbia; and in Jamaica, the latter nurse sending home no less a sum than £13 12s. 6d. Altogether 50 books were taken by nurses resident in various parts of the British Empire or abroad, the majority of which have yet to be returned, for they were only posted so recently as October 24th last. We are confident that the large sum of £2,500 collected by the nurses, and the spontaneous subscriptions of £100 a year by the policyholders will prove most gratifying to the members of the family of the late Mr. Junius S. Morgan, through whose splendid liberality and example the Royal National Pension Fund was first successfully launched. This satisfaction has found expression in the munificent donations which have been received from Mr. Morgan's eldest son, his son-in-law and daughter, all of whom take the warmest interest in the progress of the Pension Fund, and all of whom, especially Mr. and Mrs. W. H. Burns, have given so much time and thought to the heavy work entailed by its successful administration. H.R.H. the President was greatly pleased with the contributions we have the pleasure to announce, and would, we are confident, had it been possible, have gladly thanked each nurse individually for the part she rendered in raising money for those members of her profession who from no fault of their own find themselves in temporary distress, or unable to pursue their calling.

### The Humane Influence of the Modern Hospital.

By Our Special Commissioner.

Who can doubt the humane influence which the hospitals exercise upon everybody, from the physician to the patient, who is intimately associated with their work? Christmas Day, when the majority of people enjoy themselves at home, always affords a good opportunity to visit these institutions, and to see how far they fulfil the test of practice and the extent of the humane influence which they exercise. Outside the streets are often deserted and dreary, but inside the hospitals all is activity, brightness, and sociability. You enter a large ward, and are at once struck with the beauty of the decorations and the evidence of the loving care for the patients, and especially for the children, which is everywhere forthcoming. These characteristics are not confined to one ward, but are common to all. If the hospital has the advantage of being attached to a medical school, there will be found the most brilliant decorations, accompanied by every effort which the energy of youth and the best instincts of early manhood can produce to cheer the patients and make them happy and forgetful of their sufferings. The outside world little realizes the excellence and beauties which the combined efforts of students, nurses, and medical staff have introduced into hospital wards this Christmas time. A hospital is a small republic in its way, and each ward under a separate staff is as zealous for its reputation at this season as the haughtiest city on the earth's surface. The results to be seen in the wards of many hospitals this Christmas and New Year, and especially at Guy's, were well worthy of the attention and inspection of artists generally, and especially of those who devote themselves to the illustration of Christmas literature. The infinite variety of design, not only in the character but in the detail of the decorations, was remarkable. In the manufacture of ropes of evergreen alone we saw at least twenty separate designs, some of them fanciful, most of them effective, and all surprisingly beautiful. Nearly every ward contained a Christmas tree, and all were remarkable for the beauty of their flowers and the bright and attractive decorations of the tea-tables, for every sister had laid herself out to offer hospitality to the visitors who came from outside to help in the good work of making Christmas day happy and pleasant to the sick. We are confident there were no family gatherings anywhere which produced more happiness or which exercised a wider influence for good than the gatherings in the hospital wards which we had the good fortune to witness. Everybody was so friendly and pleasant that all felt at home and at peace.

During the past year a very large number of well-to-do people who have leisure at their disposal have contributed something to the support of a hospital. Very many others, a vast majority unfortunately for themselves, though well able to give, have not yet learnt the undoubted privilege thus offered them. At most of the hospitals in London and elsewhere entertainments have been given for the amusement of the patients. All classes have a craving for novelty in these days, and therefore to givers and non-givers alike a genuine surprise and real pleasure can be promised without any risk of infection at Christmas time, or, indeed, at almost any time, if they will pay a visit to some hospital. In this way not only will they benefit themselves by realising the joy to be derived from an appreciation of the actualities of the loving care lavished upon the sick, but they will undoubtedly gain a stimulus thereby which must tend to make them happier men and women than they have ever been before. They can go to a theatre or other place of amusement any day. Mystery plays, too, are becoming more popular each year, but none of these can give so much genuine joyousness to the really intelligent beholder as a spontaneous visit of inspection to the house of sickness and suffering during the Christmas season, when it is freely open to all.

Recently many families have contributed of their best to brighten the Christmas of the sick. For many hours, from one o'clock to eight o'clock, with the splendid energy and ungrudging generosity of youth, hundreds of medical students, after weeks of preparation, on Christmas Day, and by entertainments on the following days, devoted themselves to the amusement of the poor inmates of our hospitals. Some of the programmes, of which every patient had a copy, were works of art, and the talent displayed by those who took part in the various entertainments was of the best. The sisters and nurses, from daylight till long into the evening, willingly devoted themselves to the promotion of happiness and cheerfulness, for cheerfulness in the sick is the high road to recovery. So successful have these efforts been in later years that, whereas formerly every patient who could move went home for Christmas, the number of occupied beds in the hospitals this year is in many cases the largest on record. All this loving service testifies to the humane influence of the hospitals, and it will, I have no doubt, tend to excite a generous interest amongst the healthy of all classes who have it in their power to give practical expression to their gratitude—in cash. Cash or no cash, as the result of one of the happiest Christmas seasons we have ever spent, although passed with the sick in hospital wards, we confidently recommend a visit to some hospital on one afternoon or evening during the Christmas week to all who value the privilege of life as opposed to mere existence and of pleasure in contradistinction to killing time.

### Christmas in the Hospitals.

### Guy's Hospital.

At no hospital in the United Kingdom has Christmas been better kept or with greater good and satisfaction to the patients and all concerned than at Guy's Hospital. The resident staff and students deserve the grateful thanks not only of the Governors, but of everybody interested in the institution. These Christmas festivities have brought out an amount of latent talent—vocal, artistic, histrionic, and administrative—which cannot fail to prove beneficial to the governors who have so liberally rendered such a large measure of personal service in their special efforts to help the sick. The wards were charmingly decorated this year, and a thoroughly happy day was spent in the wards of this old hospital. Entertainments, in which the students played as ever an important part, continued in the various wards throughout the week, and a grand concert wound up the festivities on the 31st inst. Every movable patient was transported into Philip Ward, where a most enjoyable performance was given by (as one of the daily papers reported) "a number of ladies and gentlemen of standing on the theatrical and music-hall stages," under the direction of Mr. Charles Rily. The musical feats of the "Dis-guysed Minstrels" gave great delight.

Most of the sisters arranged for entertainments in the wards, and the festivities wound up on Saturday, the 2nd inst., with a concert held in the governors' court-room, which was organised and given by Sister Bright. "Bright Ward" is devoted to paying patients, a class not too easy to manage, and it was a distinct feather in Sister Bright's cap that so many old patients attended the concert as visitors or took part in it. Last Saturday was a day of fog, and in consequence no less than seven ladies and gentlemen who had arranged to give their services at the concert failed to put in an appearance. Most grateful thanks are therefore due to Mr. Handson, Mr. H. T. Hicks, Mr. Arthur Strugnell, and others, who threw themselves into the breach and did such excellent service. Mr. Arthur Strugnell's singing was the feature of the evening. He was formerly one of the Meister Singers, and our readers may be glad to have his address (142, Elgin Avenue, Maida Vale, W.), seeing that he is a tower of strength on an occasion like this owing to the go and finish of his vocal efforts. A concert, apart from the help which most people get from the music, is a study which may be profitably pursued by the observant. Sitting in the audience one is able to realise the differences in voice and tone and in the efforts of each performer. One may be charmed with one singer, who is succeeded by the next with a better voice, and so the hearer may become fired with envy, or afflicted with doubt in his attempt to be judicially impartial and to determine which of all the performers is the best. The artistes, too, have their compensations, for we have it on the authority of a great singer that this earth affords no greater pleasure to man or woman than the delight of singing at one's best to an appreciative and intelligent audience. In any case Sister Bright is to be heartily congratulated and thanked for her enterprise and hospitality, and her concert must ever form a pleasant memory to those who had the privilege of being present at it.

University College Hospital.

A special appeal was made this year to members of the committee and staff and friends of the hospital to carry out the usual Christmas festivities, and the result was an eminently satisfactory one. The wards were decorated, Christmas fare was provided for, and presents of warm clothing made to, all the patients; while the children had a toy-laden tree, and a very pleasant concert took place on December 30th in the out-patients' hall for all those able to be moved out of the wards. In this Mr. Hayden Coffin, Mr. C. Lovell Fry, Miss B. Lambert, the Misses Fussell, and others took part. Messrs. Maple and Co. helped in the decorations, and Messrs. Edison and Swan towards the electric lighting of the tree.

East London Hospital for Children, Shadwell.

There are few more pathetically pretty sights to be seen in London at Christmas time than the enjoyment of the children in the hospitals over the simple pleasures and gifts provided for them. The Shadwell Children's Hospital was gay with Chinese lanterns, its corridors hung with trails of ivy, and the wards bright with flowers fresh from Covent Garden on December 30th. In the fine out-patient hall a very superior Punch and Judy show held the children, grouped on the front benches and laid on their mattresses and blankets on the floor, quite entranced with pleasure, presently to be added to when the dolls and wonderful toys from two splendid trees were distributed. Realising the homes from which most of them came it was pleasant to watch their happy faces and to know that for the present, at any rate, they were blissfully forgetful of cold or hunger and entirely absorbed in the wonder of the moment. The wards at Shadwell are always charming; on Wednesday, in their festive trim, each cot with its special little bouquet, they were a sight worth seeing, and Miss Rowe and her staff must have felt well pleased with the result of their efforts.

Royal London Ophthalmic Hospital, Moorfields.

Wednesday, the 30th ult., was the day also chosen for the Christmas entertainment to the patients at the Moorfields Ophthalmic Hospital, and very successful it was. In the out-patients' hall was erected a fine Christmas tree, loaded with presents for everyone in the room, of which the distribution by the Rev. Prebendary Whittington and Mrs. Silcock caused much pleasurable excitement, and great amusement among the patients when Mr. Whittington was gravely presented with a child's toy, and Miss Robinson, the matron, with a comic doll, as their share in the gifts. A most excellent Pierrot entertainment was provided by a number of students from the London Hospital, whose clever songs were appreciated as they deserved. A particularly good sketch of the tree, and those round it, with the groups of patients, with their bandages and shades, appeared in the *Daily Graphic* of January 1st.

Royal Hospital for Women and Children, Waterloo Bridge Road.

The ever popular Punch and Judy show, a Christmas tree, and a Father Christmas, rejoiced the hearts of the small patients at the Waterloo Bridge Road Hospital on December 30th, while a concert was got up for the special pleasure of the older patients who were able to leave their beds and collect in the Alexandra Ward. The lovely toys which may be traced to the *Truth* Show were here as everywhere, and there were several toys and dolls apiece for all the children, who, lying on their beds and couches, watched Father Christmas' progress round the ward with eager anticipation. The wards were prettily decorated.

St. Mary's Day Nursery and Hospital for Sick Children, Plaistow.

Plaistow is not the easiest place in the world to reach, but those who braved the fog and found their way to St. Mary's Hospital on Saturday last felt recompensed for the difficulties of the way in seeing for themselves the pleasure of the children over their Christmas treat, their tea, and the pretty gifts from the tree, distributed by Father Christmas in regulation red robes, and hoary hair and beard. Starting only as a day nursery where the mothers of Plaistow might leave their little ones in safe and happy keeping during the day, now, in addition to the care of some thirty babies every day, the upper floor of the building has been adapted as a hospital, and contains some twenty cots. Mr. Given Wilson's exertions have resulted in this development, and now he is anxious to add a new wing for women patients. Plaistow is a long way from Shadwell or the London Hospital, yet these hospitals are the nearest, and it may be guessed in times of emergency how sorely is needed more accommodation on the spot. Miss Simmonds, the matron, was formerly a sister at the Shadwell Children's Hospital.

London Temperance Hospital.

On Christmas Day the matron of this hospital, Miss Orme, arranged a most successful concert for the patients and their friends, who were invited to tea, and to spend the evening. Madame Fortescue, L.L.C.M., most kindly gave her valued aid, bringing with her artistes whom to hear was indeed a treat. First, we must mention the talented harpist, Madame Fortescue. The splendid touch she displayed in the solos, "Within a Mile of Edinboro' Town" and "Memories," showed that she is a perfect master of her art. Miss Gleeson sang with great sweetness "Birds in the Night," whilst Senorita d'Almeida's rendering of "Rest" delighted her audience. Mr. W Walsh, a new tenor, with promises of remarkable power, was encored over and over again, and most good naturedly responded. Mr. C. W. Fleming (the mandolinist) and Miss E. Clare Gritten (pianist) each contributed their part to make the evening one likely to be long remembered by the matron, staff, and patients of the London Temperance Hospital. On December 30th a successful Christmas tree entertainment was given.

Chelsea Hospital for Women.

On Christmas Day the matron of the Chelsea Hospital for Women (Miss Mildred Heather-Bigg) arranged for her nurses a pleasant treat in the shape of a late dinner, served in the style to which most of them have been accustomed in their own homes—a welcome variation on the somewhat Spartan-like simplicity of the ordinary hospital dinner. Flowers, silver, and glass all combined to make the festive meal reminiscent of home. Another new departure in this hospital is a "Nurses' Musical Afternoon," under which modest title the nurses indicate a really capital concert, got up entirely by themselves, and given to the patients. On Boxing Day the programme included, "Oh, Who Will O'er the Downs With Me?" and several other part-songs, sung by Nurses Archer, Rolls, Burgess, Elkington, and, indeed, the whole nursing staff; a whistling song from Nurse Yessop; and Haydn's Toy Symphony, in which eight nurses, including Nurse Austin at the piano, took part. Friends of the nurses supplied the rest of the programme, Miss Harvest's violin solo being specially appreciated. The visitors and nursing staff were subsequently entertained at tea in the board-room by the matron.

### Royal Orthopædic Hospital.

As usual, at this season, the various wards were decorated for Christmas, the material for which had been kindly supplied by a member of the medical staff. On Tuesday evening, the 22nd ult., an entertainment was given to the patients and nurses by the Tudor Black and White Minstrels (members of the staff of Messrs. D. H. Evans and Co., Limited, of Oxford Street), consisting of choruses, songs, banjo solo, &c., interspersed by capital and highly amusing jokes, which caused much laughter. The programme concluded with a Christmas carol, well rendered by the whole troupe. Miss A. Percival acted as accompanist, and the piano was kindly lent by the firm. On Christmas Day each patient received a present, through the kindness of a member of the committee, and the usual Christmas fare was provided. In the evening the patients met together in the Victoria Ward and had high tea.

### The National Hospital for Diseases of the Heart and Paralysis, Soho Square, W.

Thanks to the kindness of many willing friends of the matron and resident physician, a most enjoyable Christmas Eve was spent at the above hospital. The festivities commenced with an entertainment consisting of a concert, vocal and instrumental, recitations, and musical drill. "Beauty and the Beast" was given in the second part, and the third consisted of Christmas carols, well rendered by the choir of St. Ann's, Soho. All patients that were well enough were present and appreciated to the utmost all that they saw and heard. The women's ward, where the concert took place, was tastefully decorated for the occasion. Afterwards gifts, useful and ornamental, were distributed to all patients.

### The St. Marylebone Infirmary.

The guardians and officers of the St. Marylebone Infirmary demonstrated in a most happy manner last week to a number of interested visitors how bright, comfortable, and attractive it is possible to make a Poor Law infirmary. The spirit that animates all connected with this large institution, containing over 700 poor patients, was most happily apparent to all who devoted a most pleasant and interesting afternoon to its inspection. The decorations were quite lavish and most effective in design. Those of the poor old people who were able to sit up in bed looked quaint and comfortable in their bright scarlet jackets, and evidently enjoyed the excellent tea provided for them on the occasion by the guardians. Those who were not bed-ridden clustered round the gaily-decked tea-tables in the centre of the ward. The scene was both touching and inspiring, and the remark, "We are all very comfortable here, very," came from the lips of a very old lady, who was evidently enjoying the good fare and bright scene with much zest. "If there was not so much lying in bed," she further remarked, "there would be naught to complain of." An enlightened and kindly management and excellent nursing cause the lot of the pauper patients at the St. Marylebone Infirmary to be as happy as it is possible to be.

### West Ham Hospital.

The patients at the West Ham Hospital had a very bright and cheerful Christmas, thanks to the kind support given by friends of the institution and the zealous attention of the staff. On Christmas Eve there was a large Christmas tree in the children's ward, from which every patient in the hospital had a present. The four wards were charmingly decorated with hot-house flowers and holly. On Christmas Day the good old-fashioned dinner of roast beef and plum pudding was provided. The tables were very prettily decorated, and the patients were waited on by the nurses. Christmas comes but once a year, and so it is that on that day the rules of the establishment are cancelled for a short space, and the male inmates are allowed to smoke the pipe of peace and good-will, Mr. Nathan supplying the pipes and tobacco. On Monday evening Alderman Hay, J.P. (chairman of the committee) invited all those who had been in-patients during the year to tea in the out-patients' hall, followed by an entertainment arranged by Mr. Woollright, house surgeon. "A Rough Diamond" and "The Area Belle" were very well rendered, thanks to the untiring exertions of Mr. Woollright, who for months beforehand had been working up the entertainment. To him also belonged the credit of the very successful Christmas tree.

### The Sussex County Hospital, Brighton.

The patients in this institution were not forgotten at this season, and Miss K. Scott, the matron, took much trouble in collecting subscriptions towards the Christmas entertainments. The wards were beautifully decorated, and early on Christmas morn the matron and her staff, carrying coloured lanterns, made a tour of the buildings singing Christmas carols. Each patient, in fact every person in the hospital, received a gift, in most cases consisting of something likely to be useful to them, and then came the Christmas fare for all those who were allowed to partake of it. On Boxing Day each ward had its own special tea party, and a series of entertainments were performed by the nursing staff in every ward in succession. A very pretty little stage was also rigged up in one of the wards, where a capital play was enacted before an appreciative audience of such patients as were able to be moved into the ward. A week of festivities wound up with a gigantic Christmas tree in the children's ward on Wednesday, and gave infinite delight to the poor children as well as to their elders.

### Lincoln County Hospital.

Christmas was kept in the Lincoln County Hospital as usual. The wards were decorated with evergreens and lanterns, and a tea party was given in every ward on different nights; concerts in the two male wards. In the female and the children's wards the nursing staff amused the patients by taking part in nursery rhymes in costume and various other characters. Darby and Joan created much amusement, those parts being undertaken by Nurse Laura Mills and Nurse Angell, and Britannia, by Nurse Broughton, was a great success also. Punch and Judy shows and a Christmas tree in the children's ward finished the festivities.

### City Hospital, Edinburgh.

Christmas was most happily spent in this institution. Early on Christmas morning the nursing staff sang carols in the corridors. The wards were all beautifully decorated, and in each there was a Christmas tree laden with toys and gifts. The work of decorating both these and the wards devolved on the nurses, helped by the convalescent patients. All patients who were able had turkey and other Christmas fare, and in the afternoon there was tea and games in all wards where there were no serious cases. On the day following the domestic staff were entertained to Christmas dinner and a concert, and each had several nice presents. The dinner to the nursing staff was put off, owing to the illness of the medical superintendent, but will take place next week.

Surbiton Cottage Hospital.

On Christmas Day the patients at the Surbiton Cottage Hospital were enabled to dine together in the men's ward, where they were feasted with roast turkeys (given by Mr. W. H. Roots), plum puddings (given by Mrs. Windeler), and dessert. Crackers and sweets were also provided in abundance for the children, and gave infinite delight and amusement. At five o'clock a Christmas service was conducted by the Rev. W. A. Waller, at which Mr. Leonard G. Yates, the able assistant secretary to the hospital, and about half-a-dozen boys belonging to the St. Andrew's Church choir, attended and sung a number of carols, Miss Worsley accompanying on the pianoforte. The remainder of the evening was devoted to amusements in the shape of songs, recitations, &c., the programme being entirely sustained by the nurses and patients. The wards were prettily decorated, and in the entrance hall a large motto bade the visitors "Welcome." The festivities were continued on Tuesday, when the annual Christmas tree and tea were held, to which all the children who had been in the hospital during the year—upwards of thirty-six in number—were invited. Tea was served in the men's ward, and whilst it was in progress Mr. Bristow, of Kingston, played selections on his musical bells, which were much appreciated. After tea a marionette entertainment delighted the audience, and then followed the distribution of the presents by Father Christmas.

Salisbury Union Infirmary.

The inmates of the Salisbury Union Infirmary, besides being enabled to join in the festivities of Christmas Day, were, through the kindness of the doctor, Mr. L. S. Luckhorn, and his friends, provided with another entertainment. On January 2nd, of course, it began with a tea of a most satisfactorily substantial nature, followed by a miscellaneous entertainment and distribution of tea for the women, tobacco for the men, sweets and bonbons for all, and sundry. The brightly rendered programme was made up of songs (humorous and otherwise), plantation melodies, carols, a recitation, and whistling solos, and it is needless to add that the ladies and gentlemen who took part in this concert had a very appreciative audience. Of holly and mistletoe, ivy and yew, there was an abundance and to spare, and the result was most successful. Pleasant entertainments took place on Christmas Day. The representation of an old-time maypole dance on a moss covered green, with gaily decked-out characters weaving a garland around the pole, was a great attraction, while another was "Little Jack Horner," and Charge Nurses Soper and Crane distinguished themselves and pleased their audience by excellent performances on piano and banjo. The Mayor, Mayoress, and a number of visitors present expressed much admiration at everything that had been done by the nurses for the pleasure of their patients.

Bootle Corporation Hospital.

Christmas day commenced with carol singing by the nurses. The wards were tastefully decorated with flags, banners, mottoes, monograms, mistletoe and holly, and the children's Christmas tree, laden with toys, the trunk and boughs covered with mock snow, and Jack Frost was a great success. The children were delighted, and danced round it in their glee. Santa Claus paid his usual visit at midnight to fill the stockings dangling from every patient's bed-rail, and the next morning two tiny girls, pointing up to the ventilator, solemnly declared they "saw Santy Claus flying frue in the mivvle of the night." The patients' dinner and tea were much enjoyed. In the evening the nurses organised a musical entertainment amongst themselves, which was of a varied character, and thoroughly enjoyed by all. The following Monday was a day of excitement in the wards—the day on which the Christmas tree was stripped of its treasures, every patient, as well as the staff, receiving a present. Great amusement was caused by the comical way in which "Sister" tried to quell the din when at its noisiest. She blew a horn, which gave forth such a harsh, croaking sound that it only excited renewed laughter. Altogether everyone spent a very pleasant Christmas. On January 4th the nurses gave an "At Home," which they and their friends greatly enjoyed.

Liverpool City Hospital, Grafton Street South.

The pretty wards of this hospital were tastefully decorated by the nurses for Christmas. Beautiful flowers and foliage were effectively placed everywhere. The usual sumptuous Christmas fare was provided, and toys and useful gifts were freely distributed amongst the patients in the wards. Owing to a happy thought of the matron (Miss Ilbery), instead of the usual Christmas tree, quite a variety entertainment was organised for the scarlet fever patients. There was a Punch and Judy show—the acting of which delighted the children—and afterwards a conjuror performed some very clever tricks, which met with great applause. On the Wednesday following the typhoid patients were also thoughtfully provided with an entertainment, consisting of a magic lantern of pretty views and funny pictures, followed by a concert. Everyone enjoyed themselves immensely, and spent a very happy evening. On Thursday evening the nurses held an "At Home," which was voted a success by the many guests.

York County Hospital.

Christmastide at the York County Hospital has been a very happy time. The patients were served with dinners of roast turkey and plum pudding on Christmas Day. Tobacco was supplied to the male patients, and they had the privilege of visiting the various wards and enjoying the advantages of social intercourse. There was carol singing during the day by the nurses, each of whom, it may be added, at breakfast on Christmas morn was surprised with a present on her plate. On Sunday afternoon the institution was visited by the Archbishop of York, who passed through the various wards, many of the patients as they laid in bed receiving the Archbishop's kindly greetings. On Tuesday afternoon a concert was given at the hospital by Herr Padel and the girls of the Church High School. The Christmas tree and accompanying distribution of gifts on Wednesday evening proved a great attraction. The matron's appeal had been liberally responded to, and numerous acceptable articles, besides contributions in money, came to hand. Apart from the tree, which was laden with a great variety of things, mostly, of course, suited to juvenile tastes, the presents consisted of useful clothing, greatly appreciated by the recipients. The distribution was preceded by a tea in each ward, and of the 120 patients in the hospital 90 were able to attend the proceedings in the board-room. Among those present were the Dean and Lady Emma Purey-Cust, Mr. W. H. Jalland and Mr. F. Shann, (hon. surgeons), the Rev. A. S. and Mrs. Commeline, Mrs. Trundle, Miss Gwyn (the lady superintendent), and Mr. Ashwin (house surgeon). The Dean, with the kind assistance of several of those present, distributed the gifts and gave a short address.

Reading Infirmary.

The nurses of the Reading Infirmary made the wards and corridors of the institution very pretty and attractive with decorations this Christmas.

### Dress and Uniform.

By a Matron and Superintendent of Nurses.

### THE INDIAN NURSING SERVICE UNIFORM.

Few costumes are more attractive or distinguished looking than the one adopted by the sisters of the Indian Nursing Service. The charming sketch before us depicts them in their regulation dress of light grey serge, which is made full plain white muslin without any ornamentation. The lady superintendents wear caps of a different shape to those in the sketch, which, however, are not so generally becoming. They are close fitting to the head, the material employed being white spotted muslin edged with narrow lace frills. There is also a

• OUTDOOR DRESS •

• INDOOR UNIFORM •

• WARD UNIFORM •

• SISTERS DRESS •

in front and plain at the back. Bands of military red cachemire relieve the neck, wrists, and waist most becomingly, and eight red buttons of uniform pattern fasten the bodice. In winter a grey cape with red collar is worn in the wards. A neat little "Sister Dora" cap, without strings, gives a pretty finish to the costume. It is made of slight difference in their uniform; a red waistcoat is inserted into the dress bodice, and they wear red bands at the wrists instead of on the arm. The aprons, which are of ample width, covering nearly the whole of the skirt, have two gores at the sides, good-sized pockets, and plain square bibs without straps. The lady superintendents and nursing sisters wear a similar outdoor uniform, which, as will be seen by our illustration, is as becoming as it is useful. It consists of a grey tweed ulster with smoked pearl buttons, to which is attached a grey cape and collar. The bonnet is also grey and trimmed with grey ribbon, daintily relieved by white frills and muslin strings. In summer the grey serge dress is replaced by a costume, identical in design, of cool white washing material faced with turkey-red. White sailor hats trimmed with white ribbon are permitted by the regulations to be worn out of doors; also a long circular grey alpaca cloak with hood is provided, but, needless to say, is seldom used. For evening wear dresses of soft white silk with scarlet silk facings are allowed, and a very charming appearance they present. On account of the heat grey or white pith hats or helmets have sometimes to be worn, but at present there is no uniform pattern.

### Where to Go.

TRAINED NURSES' CLUB, 12, BUCKINGHAM STREET, STRAND, W.C.—Conference of Midwives, January 15th, quarter to eight p.m. General meeting of members of the Club and Institute, January 22nd, seven o'clock. General meeting of the Society of Trained Masseuses, January 9th, half-past seven p.m.

THE NEW GALLERY.—At the New Gallery a very interesting collection of the paintings of G. F. Watts is being exhibited. Mr. Watts stands in the foremost rank of painters of our time, and it is very interesting to have this opportunity of studying so many of the most famous works of the artists, some of which were favourites of ours in previous exhibitions. The exceedingly rich tone of Mr. Watts' colouring strikes one especially when viewing his works massed together, and visitors who would fully enjoy the treat offered to them at the New Gallery should endeavour to be there before daylight fades, for Mr. Watts' love of sombre backgrounds, and environments, renders full daylight necessary, if his pictures are to receive a full meed of appreciation. We do not attempt to enter into a description of individual pictures, but we should like to draw our readers' attention to the great variety of both subject and treatment adopted by the painter. The portraits are of course of special interest. Mr. Watts is not only a great master in the branch of his art, but he has been singularly fortunate in having secured interesting sitters, and the beholder finds himself referring to the catalogue, attracted not only by admiration of the painting, but by some particular charm or interest in the countenance portrayed. In the present collection, portraits of the late Poet Laureate, Mr. Gladstone, Mary Augusta, Lady Holland, Lady Henry Somerset, Joseph Joachim, the Countess of Dudley, Sir John Millais, the Marchioness of Granby, Lord Leighton, George Meredith, the Marquis of Salisbury, and Professor Max Müller form a most interesting *ensemble*. The portrait of Lady Holland—one of Mr. Watts' earlier works—is very quaint, and forms a striking contrast to the majority of those exhibited. It was bequeathed to the Prince of Wales, and is lent by him on the present occasion. Those who have visited the National Portrait Gallery and admired the exceptionally fine specimens of Mr. Watts' works which hang on its walls, it is unnecessary to advise to go to the New Gallery, for they will assuredly do so. But those who first make the acquaintance of Mr. Watts' beautiful works at the New Gallery, where they are so well placed, should extend their pleasure by a visit to the National Portrait Gallery, where a few of his very best portraits find a fitting place.

### Pirated Advertisements.

WE have again and again had to draw attention to the unreliable character of the advertisements inserted in the *Nursing Record*, and to complain of the inconvenience caused to nurses by the conduct of the managers of that paper in copying advertisements out of our columns, and continuing to insert them after the vacancies alluded to have been filled up, thus causing annoyance both to matrons and to nurses. This we have done on public grounds, as a means of warning nurses and others whom it may concern not to rely upon notices of vacancies, &c., appearing in a paper which adopts such curious methods for puffing out its pages. The unauthorised advertisement of vacancies when no vacancies exist is a public nuisance and demands the public reprobation of reputable journals.

The *Nursing Record* of January 2nd continues its attack on Mr. Burdett. Our columns are open to him to reply if he thinks it worth while, but he may rely upon nurses as a body not being misled or carried away by the strictures of the *Nursing Record*. Mr. Burdett will decide for himself whether or not to recognise the right of the *Nursing Record* to catechise him on questions which are of no public concern.

The attack in one or two points extends to ourselves. We therefore state without qualification: (1) That Mr. Burdett has never in our columns referred to the Executive Committee of the Royal British Nurses' Association as "the scum of the nursing profession." (2) That no advertisement published in THE HOSPITAL is inserted gratuitously, and that all are paid for at ordinary rates.

Those who wish for further information on the subject of pirated advertisements may be referred to "The Mirror" for December 26th, page 115, and for December 12th, page 101, where a case is detailed and references to other cases are given.

### Canadian Nurses in the United States.

IN the *Daily Graphic* of January 5th the following cutting from a Toronto paper is printed. It refers to an order issued from Buffalo respecting Canadian nurses, and runs:—

"Buffalo, December 18th.—Immigration Inspector De Barry received from Washington last night notice of a new ruling by the Secretary of the Treasury on the Alien Labour Law. This ruling is on the question that was raised in this city two years ago as to whether the Canadian trained nurses who come to this country to work in hospitals do so in violation of the law.

"The Secretary of the Treasury has ruled that these nurses can be deported. As soon as Mr. De Barry received the notice of this new ruling he started out to get lists of the Canadian nurses in the hospitals Mr. De Barry said last night that all of these nurses would be deported.

"Inspector Estell, of Ogdensburg, is now at Dansville, where he went to deport five Canadian nurses, who are employed in a sanitarium there."

In common with the *Daily Graphic*, we can but feel incredulous that an action which exhibits such a want of reasonableness and the civilizing spirit can really be carried into effect. We can only hope that the order is an outcome of some red-tape officiousness in carrying out instructions to the letter, which, when the full bearing is understood by the more intelligent members of the administration, will be promptly cancelled, and any repetition prevented.

At the same time we have repeatedly warned nurses desirous of going to the United States that America is really as well supplied with trained women as we are in the British Isles, and that alien nurses are consequently as little likely to be welcomed there as they would be here. It rests with the hospitals in the United States to make rules to the exclusion of foreign nurses if they so desire to do, but those that are already engaged should certainly not be removed from office in the arbitrary way the paragraph indicates. No doubt the authorities of the great hospitals and training schools in the United States are too sensible of the debt of gratitude they owe to Canadian nurses to permit them to countenance, much less to act upon, any order similar to that issued from Buffalo.

### Everybody's Opinion.

[Correspondence on all subjects is invited, but we cannot in any way be respn sible for the opinions expressed by our correspondents. No communication can be entertained if the name and address of the correspondent is not given, or unless one side of the paper only is written on.]

### HELP WANTED.

Nurse Adelaide Bourne, Union Infirmary, Wilton, whose letter asking help through The Hospital towards giving her patients a Christmas treat was published under this head in the Christmas number, writes: Now our Christmas festivities are over, I must ask you to be kind enough to allow me to thank, through your columns, all those kind friends, including yourself, who have so generously responded to my appeal through The Hospital. Amongst the visitors to the wards on Christmas afternoon were the Countess of Pembroke (her first visit to the wards), Lord Herbert of Lea, Lady Muriel Herbert, Mrs. Vanderbilt, the Misses Naish, Bagnall, and Rawlence, Dr. Straton, the Master and Matron, and others. Through the generosity of friends at Wilton, South Newton, and Burcombe, and through readers of The Hospital, I was enabled to give to each woman a present and also to the boys. Lady Pembroke distributed the presents on her arrival, stepping over the forms to give the old women in bed their pretty flannelette jackets (one old lady was 85, the other 83); the others had pocket handkerchiefs. The old men were given ½ lb. tobacco and a pipe each, the able women, ¼ lb. of tea and a handkerchief, while the children had toys of all descriptions. The children then sang, "While Shepherds Watched their Flocks by Night," afterwards giving three lusty cheers for Lady Pembroke and all the kind friends who had given them such a treat. The lady visitors sang some pretty carols, which were much appreciated by the old folks, some saying they had never "heard the like"; one old woman who has been in the house about ten years says she "had never seen such a sight here before." They all send you their very grateful thanks, and wish you a very happy New Year. Will you please acknowledge the following kind contributions to our treat, obtained through your readers? Miss Duckworth, 2s. 6d.; Sister White, 2s. 6d.; Mr. Hans Willeringhaus, cocoa; Miss Salt, books; Miss J. Wilson, toys; Miss Erle, 10s.; Mrs. and Miss Braid, toys, &c.; Friends at Kettering, large box of toys, and money; from Earl's Court, parcel of crackers; from Bideford, toys and packets of tea; Miss G. M. Godden, 5s.

⁂ Few things have given us more pleasure this Christmas than to have thus been the means, by a little private exertion as well as through the publication of Nurse Bourne's letter, of helping her to give the poor people under her care this small measure of happiness. Through a friend it was possible to arouse local interest in the Wilton Union, which we hope will continue to flourish not only at Christmas time but throughout the year, and that Nurse Bourne may in future find all the kindly help she needs close at hand.—Ed. *T. H.*

### MENTAL NURSES AND THE ROYAL BRITISH NURSES' ASSOCIATION.

Miss L. C. East, M.R.B.N.A., late Lady Superintendent of the National Hospital for Paralysis and Epilepsy, Queen Square, London, W.C., late Member of the Executive Committee of the General Council of the R.B.N.A., writes: On reading Miss S. G. Wingfield's letter in the *Morning Post*, and also in a recent issue of the *Nursing Record*, I find it most misleading both to members of the R.B.N.A. and also to the general public, to whom it appeals for support. Miss Wingfield states that the scheme for admitting untrained mental nurses, or rather, as she puts it, "those who have had no training in a general hospital," has not had the sanction of any but the honorary officers of the association. This is not correct, as the scheme for admitting properly trained and qualified mental nurses holding the pass certificate of the Medico-Psychological Association was adopted by the General Council of the Association at a meeting of which Miss Wingfield received due notice, and could have attended and voted against the scheme with which she says she is not in sympathy. This she certainly did not do, for the scheme was carried unanimously. Judging from the letters of T. Outterson Wood, M.D., and S. A. K. Strahan, M.D., in the *British Medical Journal* of December 19th, these trained mental nurses have to pass through quite as severe a three years' course of training, and pass examinations in elementary anatomy and physiology and the care and management of the sick, as is enforced in any general hospital. This attack on the mental nurses holding their certificates of three years' training is nothing but self-interest on the part of a small faction of the Association, who, in a narrow-minded fashion, founded on gross ignorance of the claims of mental nurses to be registered, are endeavouring to stir up opposition to what is only an act of justice to them and to the public, who, by their registration, would be enabled to judge of the training and status of the mental nurses they are, by necessity, called upon to engage. It is proposed that the mental nurses shall be put on the register as mental nurses of the Royal British Nurses' Association—M.N.R.B.N.A.—thus stamping them with their special qualification. As our Charter grants us the right to admit such members, I, with many other matrons of experience, see no reason why we should not gather to the Association as many trained nurses as we possibly can. I, with those who have had experience in acute mental and nervous diseases, know (with rare exceptions, which are remarkable) how little use the ordinary trained hospital nurse is to cope with acute mental cases, or even certain forms of nervous disorder that are admitted either into general hospitals or special hospitals for nervous diseases, in the hope that the attack may be only a passing one that will, and does, give way to special treatment. The strain on the nurse who is not trained in mental work is too severe, and she has not the same influence over her patient. From what I know of general hospital nurses, they, as a rule, shirk taking mental cases because of their inexperience of such cases. Therefore, is it not only right and just that these qualified mental nurses should be accepted on the register of the R.B.N.A., so that the general public can be sure of engaging those who understand the nursing of the mentally-afflicted sick?

### Appointments.

### MATRONS.

Government Hospital, Accra, Western Africa.—Miss Hanley, who has been appointed Matron of this hospital, was trained at the Brownlow Hill Infirmary, Liverpool, and she afterwards joined the staff of the Nurses' Home, Granville Road, Newcastle-on-Tyne, where she has remained for five and a half years. Miss Hanley and the nurse who will accompany her are the first English nurses sent out to Accra, where the hospital is a new institution. Miss Hanley sails on the 16th inst., and we wish her a pleasant voyage and successful career.

North Western Fever Hospital.—Miss Maud Mary Lloyd has been appointed Matron of the above hospital. She was trained at St. Thomas's Hospital, held the posts of head nurse and night superintendent at the St. Marylebone Infirmary, and was assistant matron at the Royal Infirmary, Liverpool.

### Presentations.

The nurses of the City Hospital, Walker Gate, Newcastle-upon-Tyne, have presented their much-esteemed matron (Miss Ogston) with a very handsome album as a New Year's gift.—On Christmas morning the nurses of the South Wales Nursing Co-operation, Cardiff, presented Miss Mackie, the lady superintendent, with a handsome pair of engravings in oak frames as a token of their esteem and also of their gratitude for the efficient manner in which the co-operative home has been worked since it was started in 1894. Miss Mackie, in thanking them for their kind thought of her, assured them that the financial condition of the institute was satisfactory, and that she hoped each year would find them increasing in numbers, and winning further the confidence of the public.

### For Reading to the Sick.

THE GREAT LESSON OF OBEDIENCE.—EPIPHANY.

"THOUGH He were a son, yet learned He obedience."—*Heb.* v. 8.

**Verses.**

The Heavenly Child in stature grows,
And, growing, learns to die;
And still His early training shows
His coming agony.

He, whom the choirs of Angels praise,
Bearing each dread decree,
His earthly parents now obeys
In deep humility.

The Perfect Way is hard to flesh,
It is not hard to love;
If thou wert sick for want of God
How swiftly would'st thou move!

Be docile to thine unseen Guide,
Love Him as He loves thee;
Time and obedience are enough,
And thou a Saint shall be. —*Faber.*

I would not have the restless will
That hurries to and fro,
Seeking for some great thing to do
Or secret thing to know;
I would be treated as a child,
And guided where I go.

In a service which Thy will appoints
There are no bonds for me!
For my inmost heart is taught the truth
That makes Thy children free;
And a life of self-renouncing love
Is a life of liberty! —*A. L. Waring.*

To prayer, repentance, and obedience due,
Though but endeavoured with sincere intent,
Mine ear shall not be slow, mine eye not shut.
—*Milton.*

God's will on earth is always joy,
Always tranquility. —*Faber.*

**Reading.**

If ye be willing and obedient, ye shall eat the good of the land.—*Isa.* i. 19.

By the obedience of one, shall many be made righteous.—*Rom.* v. 19.

The whole course of things goes to teach us faith. We need only obey. There is guidance for each of us, and by lowly listening we shall hear the right word.—*Emerson.*

He was asking questions, Who was in Himself the sole sufficient answer to all questions that could be asked. He was seeming to learn in order that He might more sweetly teach. Surely in another moment He will appear as confessed, undoubted God. The angels remember Him as He was at that wondrous moment; to Mary's love and Joseph's faith manifest God, to the others a wonder, a potent, an enigma, yet to all of them a not unchildlike Child.

—*F. W. Faber.*

"It is difficult for us to understand that men engaged in the little affairs of this poor unsatisfying life on earth, with all its petty concerns and troubles, are what Scripture reveals to us, heirs of immortality intended for heaven, to be made equal to the angels and to dwell for ever with God. And yet our blessed Saviour would not only have us deeply impressed with this truth ourselves, and always acting under this impression, but also to look upon others in this light as fellow-heirs of the grace of life. Our Lord's childhood at once raises the common life of us all up to heaven."—*Isaac Williams.*

### The Book World for Women and Nurses.

MESSRS. ABBOTT, JONES, AND CO. announce for next week "The Narrative of My Experience as a Volunteer Nurse in the Franco-German War of 1870-1," by Anne Thacker, with a sketch of her life by James M. Menzies, M.A., with two photographs.

### Notes and Queries.

The contents of the Editor's Letter-box have now reached such unwieldy proportions that it has become necessary to establish a hard and fast rule regarding Answers to Correspondents. In future, all questions requiring replies will continue to be answered in this column without any fee. If an answer is required by letter, a fee of half-a-crown must be enclosed with the note containing the enquiry. We are always pleased to help our numerous correspondents to the fullest extent, and we can trust them to sympathise in the overwhelming amount of writing which makes the new rules a necessity. Every communication must be accompanied by the writer's name and address, otherwise it will receive no attention.

*Nursing.*

(81) 1. Please tell me of a good book, not elementary, on medical and surgical nursing. 2. Would "Buck's Vest-Pocket Dictionary" (Baillière, Tindall, and Cox) be suitable in place of the "Nurses' Dictionary," as I often want a more comprehensive one?—*Marie.*

1. Miss Isabel Hampton's "Nursing," or the new edition of Miss C. Weeks-Shaw's "Text Book of Nursing" should suit your purpose. You can obtain both from the Scientific Press, 28 & 29, Southampton Street, Strand, W.C. 2. "Buck's Dictionary" is a very good little book, but relates to medical rather than purely nursing matters.

*Unsuitable Questions.*

(82) A correspondent writes to ask us to "describe a real Jubilee half-crown."

We must point out to "Nurse G." and to other correspondents that the space at disposal for replies to queries is very limited, and that some often have to be held over for several weeks. In these circumstances nurses will help us by keeping their queries to professional matters as nearly as possible. Such a query as that of Nurse G.'s would best be answered by one of the many ladies' papers.

*Cruelty to Animals.*

(83) Can you tell me if the statement recently made by "Ouida," that the skin used in the manufacture of Suède gloves is taken from the living calf is true or not?

Many people are anxious to know if this is a true indictment, and inquiries are being made on the subject, the result of which will duly appear in THE HOSPITAL.

*L.O.S. Examination.*

(84) Is it possible to pass the L.O.S. examination with only three months midwifery training, and having had no other experience in the work? What is the fee?

Certainly it is possible, but it would be far better to obtain at least a year's training in general nursing first, if you intend taking up regular employment as a midwife. The examination fee is £1 1s. You can obtain all particulars from the Secretary of the L.O.S., 20, Hanover Square, W.

*Poor Law Officers' Superannuation.*

(85) Please tell me what Miss Wilson meant by saying she would second Mr. Rutherglen if he would get the other Bill carried by the Local Government Board? That was in the report in THE HOSPITAL of a meeting held by the Workhouse Nursing Association on the subject of Poor Law Superannuation for Nurses. What is the Bill?

You do not seem to have read the report of the meeting in question at all carefully. In the first place, the meeting was held by the Hospitals Association, not the W.I.N.A. The Superannuation Act now in force compels Poor Law nurses to contribute to a fund from which they are most unlikely to gain any benefit. As this is considered by many an injustice, an Amendment Bill has been drafted, which will enable nurses to choose whether they shall contribute to the Superannuation Fund or not. It is most important for Poor Law nurses who desire that this Amendment Bill, which will protect their interests, should become law, to write to that effect to the Hon. Secretary, Petition Committee, 27, Percy Street, W.

*The Nurse Dolls.*

(86) Could it be arranged for the Nurse Dolls to give an exhibition in aid of the Junius S. Morgan Benevolent Fund, to swell the yearly contributions from members of the Pension Fund?—*Every little makes a muckle.*

We fear this cannot be managed; such an exhibition would not be sufficiently generally attractive to be worth getting up. It would involve a good deal of trouble and expense, and the profits could be but small.

*Quarantine.*

(87) If a nurse nursing scarlet fever disinfects with the patient for ten days, will she at the end of that time be safe and free to go amongst other people?—*Infectious.*

It is not at all desirable for a nurse to go straight from a convalescent fever patient to mix with other people. She should take a few days' further quarantine. Private nurses are often in difficulty on this matter as to where to go. Perhaps you do not know that Miss C. J. Wood, Nurses' Hostel, Percy Street, W.C., takes nurses for quarantine after nursing infectious cases.

## Figures and Tables

**Fig. 1. f1:**
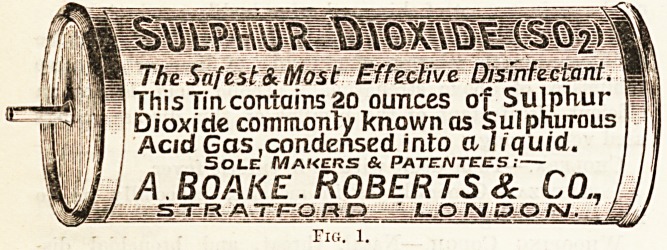


**Fig. 2. f2:**
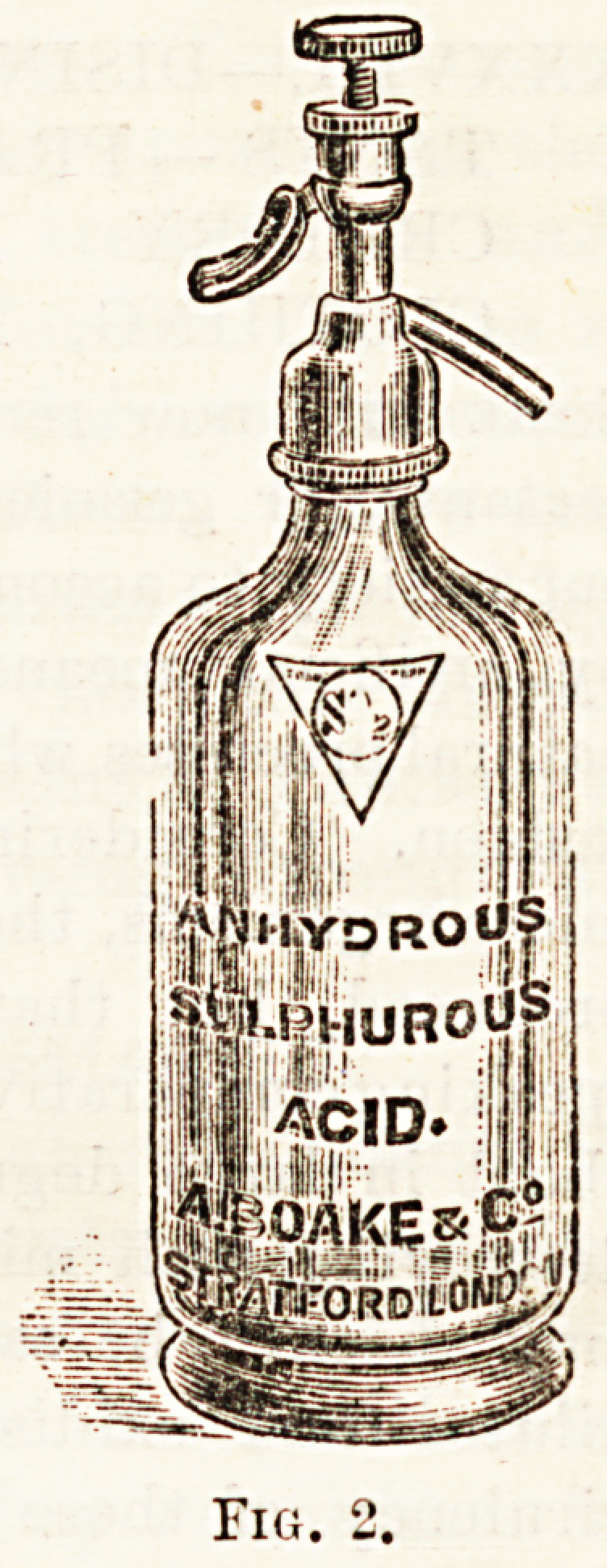


**Figure f3:**